# Insights and perceptions: Investigating pregnant women’s attitudes, understanding, and factors influencing knowledge regarding medication usage during pregnancy—A cross-sectional study

**DOI:** 10.1371/journal.pone.0311235

**Published:** 2024-10-01

**Authors:** Abdullah R. Al-khawlani, Qutaiba A. Qasim, Abdulsalam M. Halboup, Samar Thiab, Mohammed Zawiah, Fahmi Y. Al-Ashwal

**Affiliations:** 1 Department of Pharmacy, College of Medical Sciences, AL-Saeeda University, Dhamar, Yemen; 2 Institute of Pharmaceutical Chemistry, Philipps University of Marburg, Marburg, Germany; 3 Department of Clinical Pharmacy, College of Pharmacy, Al-Ayen Iraqi University, Thi-Qar, Iraq; 4 Department of Clinical Pharmacy and Pharmacy Practice, University of Science and Technology, Sana’a, Yemen; 5 Discipline of Clinical Pharmacy, School of Pharmaceutical Sciences, Universiti Sains Malaysia, Penang, Malaysia; 6 Faculty of Pharmacy, Applied Science Private University, Amman, Jordan; 7 Department of Clinical Practice, College of Pharmacy, Northern Border University, Rafha, Saudi Arabia; Universiti Sains Malaysia, MALAYSIA

## Abstract

**Background:**

Medication use during pregnancy is a critical concern due to potential risks to both the mother and fetus. To the extent of our knowledge, there has been no prior research to assess medication use and identify the specific factors of pregnant women within the Yemeni community. This study aimed to investigate the knowledge, beliefs, and practices of Yemeni pregnant women regarding medication use and assess the factors associated with the knowledge during pregnancy.

**Methods:**

A cross-sectional multi-center study was conducted through face-to-face interviews using a validated structured questionnaire. The study was conducted on pregnant women receiving antenatal care at tertiary care hospitals in four governorates in Yemen. Univariable and multivariate logistic regressions were employed to examine the relationship between participant variables and their knowledge. A *P*-value of less than .05 was considered statistically significant.

**Results:**

Out of the 1003 pregnant women, 35.4% (n = 355) were aged 21–25 years, 33.9% (n = 340) had primary education, 73.6% (n = 738) lived in urban areas, 12.2% (n = 122) were smokers, 38.3% (n = 384) reported chewing khat, and the majority (90.2%, n = 905) were unemployed. Also, 65.5% (n = 657) of the participants reported taking folic acid in their current pregnancy. A total of 2,623 medications were utilized during pregnancy, with 17.8% during the first trimester (n = 468). Around 39.3% (n = 1,037) of medications used fell under the blood and blood-forming organs category. Education level (AOR: 4.00, *P* < .001), insurance status (AOR: 1.71, *P* = .026), information about medication risks to the fetus (AOR: 1.96, *P* = .023), the use of folic acid either in a previous pregnancy (AOR: 1.65, *P* < .008) or in the current pregnancy (AOR: 4.26, *P* < .001), and checking the medication leaflet (AOR: 5.67, *P* < .001) were predictors of higher knowledge.

**Conclusion:**

The findings underscore the pressing need for educational interventions aimed at pregnant women. By bridging knowledge gaps and promoting informed decision-making, such initiatives can contribute to a safer and healthier pregnancy journey, reducing the inclination towards self-medication.

## Introduction

Medication use among pregnant women has increased remarkably [1, 2], whether prescribed or over-the-counter (OTC) [3], driven by increasing preexisting medical conditions and the emergence of pregnancy-related medical issues [3–5]. Despite some women being well-informed about high-risk medications during pregnancy, a prevalent “general fear” surrounding medication use persists [6], potentially leading to severe repercussions, including avoidance of nausea and vomiting treatments [7], non-adherence to prescriptions [8], a preference for herbal remedies [9], OTC use [10], and various self-medication practices [11].

One of the essential vitamins that pregnant women should take is folic acid. It is a water-soluble vitamin that is essential for the development of the fetus’s neural tube. The current recommendations state that all women who are planning or could become pregnant should take 400–800 *μ*g of folic acid daily, ideally for at least one month before conception and throughout the first trimester of pregnancy [12, 13].

Medication use during pregnancy has been investigated across populations. For instance, Norwegian women have exhibited a generally positive outlook toward medication but tend to be more cautious during pregnancy [14, 15]. In Malaysia, many pregnant women had negative beliefs and insufficient knowledge regarding the potential risks associated with medication use during pregnancy [16]. In Tanzania, a significant proportion of pregnant women reported hesitating to take medications without consulting their physicians, and only a minority were aware of specific drug contraindications during pregnancy [17]. In addition, the majority of pregnant Italian women recognized the risks of using unprescribed medications during pregnancy and preferred medical advice. They would not self-medicate without a doctor’s prescription but might for non-serious issues, with a pharmacist’s recommendation, or emergencies [18].

Despite these insights, there is a significant gap in the literature regarding the specific context of Yemeni pregnant women, whose health practices may be shaped by unique cultural, socioeconomic, and healthcare access factors. To the extent of our knowledge, there has been no prior research to assess medication use and identify the specific factors of pregnant women within the Yemeni community that impact awareness about medication usage. This study holds significance due to reports indicating increased self-medication rate among pregnant women, even for medications that should strictly require prescriptions and limited knowledge of safety implications associated with self-medication practice [19]. Understanding these factors is crucial to inform public health strategies, education campaigns, and healthcare provider guidance that are tailored to the needs and circumstances of Yemeni women. This study aimed to investigate the knowledge, beliefs, and practices of Yemeni pregnant women regarding medication use and assess the factors associated with the knowledge of medication use during pregnancy.

## Methods

### Study design, setting, and population

A cross-sectional study was conducted over a period of three months between April 2023 and June 2023 on pregnant women receiving antenatal care at tertiary care hospitals in four governorates in Yemen. These include Sana’a governorate (the capital city), Dhamar governorate, Ibb governorate, and Al-Bayda governorate. These hospitals that were visited have specialized units for the specialized care of pregnant women and serve both governorates’ residents and people from other countryside. Moreover, these centers offer various services for pregnant women, such as in-center ambulatory, inpatient, and outpatient services, providing pregnant women with the necessary knowledge. The study included women with confirmed pregnancy, aged ≥ 18 years, and seeking antenatal care at the selected hospitals. Patients with mental illness, dementia, critically ill, or who did not provide consent were not eligible to participate in the study‏.‏

### Sample size calculation

We used Daniel’s sample size formula to determine the sample size, with a 95% confidence level, 5% margin of error, and 50% response distribution [20]. The required minimum sample size was calculated to be 385 pregnant women. Adding a 20% non-response rate and in case of incomplete data, the total sample size was 481. A non-probability convenience sampling technique was used to enroll participants. Eligible pregnant women attending the prenatal clinics were approached, and those who declined to participate were excluded from the study‏.

### Study tools and data collection

Trained pharmacy students administered the questionnaire in person via an interview-based approach, simplifying the language and clarifying the questions as needed to ensure that participants could fully comprehend and respond accurately. This method allowed participants to convey their responses verbally, ensuring that illiteracy did not prevent them from being included in the study. The questions were adopted from previous literature [18, 21]. Three experts in clinical pharmacy assessed the content relevance, appropriateness, and representation of the evaluated tool, and modifications were made to fit the Yemeni setting. Also, a pilot study among 39 pregnant women was done to assess the clarity of the questionnaire. The alpha Cronbach of the knowledge was 0.760.

The questionnaire consisted of four sections. The first section, demographic data, collected information on demographic variables such as age, marital status, education, income, insurance, occupation, smoking, and khat chewing status. Also, the first section contained questions about gravidity and parity, including whether the participant had experienced any incomplete pregnancies or had chronic medical issues. Additionally, we inquired about their habit of checking medication leaflets before pregnancy, as well as whether they had engaged in preconception planning by visiting a doctor. Furthermore, the questionnaire addressed their current gestation status and the frequency of their visits to the gynecologist.

The second section, knowledge, assessed the participants’ knowledge and understanding of medication use during pregnancy. Questions covered important aspects related to medication and folic acid awareness during pregnancy. Participants were asked if they were aware that certain medicines might not be safe during pregnancy, if they knew about the necessity of taking specific medications during this period, and if they were familiar with the critical period during pregnancy when medications have the most significant impact on the fetus. The subsequent questions focused on participants’ knowledge of folic acid, their awareness of neural tube defects, and the potential relationship between folic acid deficiency and these malformations. Lastly, participants were questioned about the optimal timing for commencing folic acid, ensuring that they had a grasp of this critical prenatal supplement’s importance and timing.

In the third section, the questionnaire delved into participants’ beliefs concerning medication use during pregnancy. Participants were presented with a series of statements to which they could express their agreement or disagreement on a Likert scale. The statements encompassed a spectrum of perspectives on medication use during pregnancy. This includes "all medications can be harmful to the fetus", "it is best for the fetus if the pregnant woman stops taking medications during pregnancy", "it is better for the fetus if pregnant women use medications for treatment instead of leaving the condition untreated", "pregnant women should consult any healthcare provider before using natural remedies", and "it is necessary to consult a gynecologist before taking any medication".

The fourth section, practice, assessed the participants’ self-reported medication use during pregnancy, including the types of medications taken, the frequency of use, and the sources of these medications. For instance, the questionnaire explored whether pregnant women habitually reviewed medication leaflets and safety information. Additionally, participants were asked if they informed pharmacists about their pregnancy when buying medications. The questionnaire then delved into prescription medication usage during the current pregnancy, inquiring about the number of prescribed medications being taken. Transitioning to non-prescription medication practices, participants were asked if they had used any non-prescribed medications during their current pregnancy, and the number of such medications was specified. The questionnaire also touched upon familiarity with and use of folic acid, questioning whether participants had utilized it in previous pregnancies and if they were currently taking it. Also, participants were asked about the timing of folic acid initiation in the present pregnancy.

### Ethical approval

The ethical approval was obtained from the medical ethics committee at Al-Saeeda University, Dhamar, Yemen (IEC/SRC/SU/2023/EX. 21/009/23). As the study did not involve any medical intervention or invasive procedures and there were no anticipated risks for the participants, the verbal informed consent was approved by the committee. All data was collected anonymously.

### Statistical analysis

The data were imported from an Excel sheet into IBM SPSS Statistics version 21.0 for Windows (IBM Corp., Armonk, NY, USA). Categorical variables, including gender, pregnancy status, and education level, were presented as frequencies and percentages. In terms of knowledge-related questions, a scoring system was implemented where a score of 1 was assigned for each correct answer and 0 for each incorrect. The overall knowledge was then categorized using Bloom’s cut-off point into "good knowledge" if the participants’ responses were ≥ 80% and "poor knowledge" if their responses were < 80% [22]. Univariable logistic regression was employed to examine the relationship between participant variables and their knowledge of medication use during pregnancy. Variables with a *P*-value of less than 0.25 in the univariable logistic regression were exposed to multivariate logistic regression to build a predictive model for participants’ knowledge about medication use. Odds ratios were calculated to quantify the impact of each predictor on the overall knowledge. A *P*-value of less than 0.05 was considered statistically significant.

## Results

### Participants’ sociodemographic characteristics

The sociodemographic characteristics of the participants showed that nearly one-third (35.4%, n = 355) of the participants fall within the age range of 21–25 years, while education level revealed that 33.9% (n = 340) have primary education. In terms of residence, a substantial portion (73.6%, n = 738) lives in urban areas, and the majority (90.2%, n = 905) are unemployed, with 53.1% (n = 533) reporting a monthly income of ≤ 55000 Yemeni Riyals (YER) (One US Dollar = 550 Yemeni Riyals).

In terms of social habits, 54.4% (n = 546) reported chewing khat, and 20.9% (n = 210) smoked before pregnancy. Regarding past medical history, 29.6% (n = 297) disclosed having chronic diseases. Intriguingly, 44.5% (n = 446) are in the third trimester of pregnancy, and 76.4% (n = 766) did not seek preconception planning from a doctor. Moreover, the majority (70.2%, n = 704) of the participants were parous, and a proportion of 36% (n = 361) of women reported a monthly follow-up with a gynecologist. Other details are shown in Table 1.

**Table 1:** Participants’ sociodemographic data (n = 1003).

		Count	(%)
Age (years)	18–20	225	(22.4)
	21–25	355	(35.4)
	26–30	245	(24.4)
	31–35	110	(11.0)
	>35	68	(6.8)
Education level	illiterate	152	(15.2)
	Primary	340	(33.9)
	Secondary	149	(14.9)
	High school	187	(18.6)
	Diploma or higher	175	(17.4)
Living place	Rural	265	(26.4)
	Urban	738	(73.6)
Governorate	Sana’a	421	(42.0)
	Dhamar	343	(34.2)
	Ibb	98	(9.8)
	Al Bayda	141	(14.1)
Income	≤55 thousand	533	(53.1)
	55-less than 110	258	(25.7)
	≥110 YR	212	(21.1)
Job	Unemployed	905	(90.2)
	Employed	98	(9.8)
Smoking before pregnancy	Yes	210	(20.9)
	No	793	(79.1)
if yes, does you smoke during the current pregnancy	Yes	122	(57.0)
	No	92	(43.0)
Khat chewing before pregnancy	Yes	546	(54.4)
	No	457	(45.6)
if yes, does you chew khat during the current pregnancy	Yes	384	(69.9)
	No	165	(30.1)
Insurance	No	890	(88.7)
	Yes	113	(11.3)
Gravidity	≤ 3 times	628	(62.6)
	> 3 times	375	(37.4)
Parity	Nulliparous	299	(29.8)
	Parous	704	(70.2)
Have you experienced none completed pregnancies?	Yes	347	(34.6)
	No	656	(65.4)
Do you have chronic diseases or any medical issues? *	No	706	(70.4)
	Yes	297	(29.6)
Before pregnancy, do you usually check the medication leaflet for information about the medications you use?	Yes	337	(33.6)
	No	423	(42.2)
	Sometimes	243	(24.2)
Did you visit your doctor before pregnancy for preconception planning?	No	766	(76.4)
	Yes	237	(23.6)
Current gestation	First trimester	240	(23.9)
	Second trimester	317	(31.6)
	Third trimester	446	(44.5)
How often do you visit your gynecologist?	Every two weeks	73	(7.3)
	Every 4 weeks	361	(36.0)
	Every 6 weeks	114	(11.4)
	Every 8 weeks	178	(17.8)
	More than 8 weeks	276	(27.5)

*Chronic diseases included anemia, hypertension, chronic kidney disease, diabetes mellitus, dyslipidemia, peptic ulcer, cardiovascular disease, asthma/chronic obstructive pulmonary disease, migraine, hypo/hyperthyroidism.

### Medication use during pregnancy

Table 2 reveals the pattern of medication use during pregnancy among the study participants. In terms of awareness and safety, more than half (56.5%, n = 567) of the participants reported checking the leaflets and safety information of a medication prior to use it, and the majority (90.6%, n = 909) informed pharmacists about their pregnancy when purchasing medication. Moreover, 84.8% (n = 851) of the participants reported using prescription-only medication during their current pregnancy.

**Table 2:** Use of medications during pregnancy (n = 1003).

		Count	(%)
During pregnancy, do you usually check the leaflets and safety information of the medication you use?	No	436	(43.5)
	Yes	567	(56.5)
Do you inform the pharmacist that you are pregnant when purchasing any medication from the pharmacy?	Yes	909	(90.6)
	No	94	(9.4)
Do you take any prescribed medications from your doctor during the current pregnancy?	Yes	851	(84.8)
	No	152	(15.2)
POM	0	150	(15.0)
	1	178	(17.7)
	2	245	(24.4)
	3	209	(20.8)
	4	109	(10.9)
	5	61	(6.1)
	6	51	(5.1)
Have you used any medication during pregnancy without a prescription from your doctor?	Yes	250	(24.9)
	No	753	(75.1)
Number of OTC medication	One medication	196	(80.3)
	≥ 2 medications	48	(19.7)
Have you used folic acid in previous pregnancies?	No	434	(43.3)
	Yes	569	(56.7)
Are you currently taking folic acid?"	No	346	(34.5)
	Yes	657	(65.5)
In your current pregnancy, when did you start using folic acid?	During preconception planning	53	(5.3)
	after discovering you are pregnant	372	(37.1)
	First trimester	291	(29.0)
	Second trimester	49	(4.9)
	Third trimester	4	(0.4)
	I still don’t use it."	234	(23.3)

In terms of the prescribed medications, 24.4% (n = 245), 20.8% (n = 209), and 17.7% (n = 178) took two, three, and one agent, respectively. Moreover, 24.9% (n = 250) of the participants acknowledged using non-prescribes medications during their pregnancy, with a large proportion of them (80.3%, n = 196) reporting using a single medication. In comparison, 19.7% (n = 48) reported using two or more.

Regarding the practice of using folic acid during pregnancy, over half of the participants (56.7%, n = 569) have used folic acid in previous pregnancies. Currently, 65.5% (n = 657) of the participants reported taking folic acid in their ongoing pregnancy. When it comes to the timing of taking folic acid, the majority (37.1%, n = 372) began taking folic acid after discovering their pregnancy, followed by 27.0% (n = 291) in the first trimester. A smaller proportion (5.3%, n = 53) started taking folic acid during preconception planning. Interestingly, 23.3% (n = 234) of the participants still did not use folic acid.

### Knowledge and awareness of medications use during pregnancy

The participants’ knowledge and awareness regarding medication use during pregnancy are presented in Table 3. The overall median knowledge score across the assessed questions was 8 (IQR: 4), indicating variability in participants’ understanding. The majority of participants (83.0%, n = 832) had an understanding that certain medications might not be safe during pregnancy, while others are important (79.0%, n = 792). Similarly, a significant proportion (65.8%, n = 660) demonstrated awareness of the critical period during pregnancy when medication could have negative consequences on outcomes. Moreover, a majority (86.5%, n = 868) knew that medications for chronic conditions must be adjusted before pregnancy.

**Table 3:** Participant’s knowledge and awareness regarding medications use during pregnancy.

Questions		Count	(%)
Are you aware that some medications may not be safe during pregnancy?	No	171	(17.0)
	Yes	832	(83.0)
Are you aware that some medications are important during pregnancy	No	211	(21.0)
	Yes	792	(79.0)
Do you know what the critical period during pregnancy is, where medications may have a greater impact on the fetus?	No	343	(34.2)
	Yes	660	(65.8)
If the answer is yes to the previous question, please specify the time period.	Incorrect answer	35	(5.3)
	Correct answer	625	(94.7)
Is it recommended to adjust medications for chronic conditions before pregnancy?	No	135	(13.5)
	Yes	868	(86.5)
Have you heard about folic acid?	No	125	(12.5)
	Yes	878	(87.5)
Are you aware of the role of folic acid during pregnancy?	No	243	(24.2)
	Yes	760	(75.8)
Have you heard about neural tube defects?	No	917	(91.4)
	Yes	86	(8.6)
Is there connection between folic acid deficiency and congenital diseases?	No	824	(82.2)
	Yes	179	(17.8)
What is the appropriate time to start taking folic acid supplements?	Incorrect answer	226	(22.5)
	Correct answer	777	(77.5)
What is the appropriate prophylactic dose of folic acid?	Incorrect answer	349	(53.0)
	Correct answer	310	(47.0)
Overall awareness and Knowledge, Median (IQR): 8(4)			

Abbreviations: NTD: Neural Tube defects; IQR: interquartile range

Additionally, a substantial number of participants were familiar with folic acid (87.5%, n = 878) and its role during pregnancy (75.8%, n = 760). However, only a small percentage (8.6%, n = 86) had heard about neural tube defects, and just 17.8% (n = 179) were knowledgeable about the association between folic acid deficiency and neural tube defects. Concerning the timing and dosage of folic acid supplementation, 77.5% (n = 777) knew the recommended timing, and 47.0% (n = 310) were aware of the appropriate prophylactic dose during pregnancy.

### Associations between participants’ sociodemographic characteristics and their knowledge about medication use during pregnancy

In the univariable analysis outlined in Table 4, significant associations were observed between participants’ sociodemographic factors and their pregnancy medication knowledge. Notably, participants aged >25 years exhibited significantly higher knowledge (COR: 1.65, *P* = .001), as did those with a diploma education or higher (COR: 6.67, *P* < .001), and urban residents (COR: 2.79, *P* < .001). Moreover, participants who have higher income levels (>55,000 YER) were also associated with better knowledge (COR: 2.46, *P* < .001), along with employment (COR: 2.84, *P* < .001), insurance status (COR: 3.57, *P* < .001), and gynecologist visit frequency (COR: 2.00, *P* < .001), preconception planning (COR: 1.6, *P* = .003), chronic diseases or any medical issues (COR: 0.672, *P* = .016), previous use of folic acid (COR: 2.38, *P* < .001), current folic acid use (COR: 6.54, *P* < .001), and checking the medication leaflets (COR: 5.67, *P* < .001). However, factors like gravidity and completed pregnancies did not maintain significant associations.

**Table 4:** Association between participants’ sociodemographic data and their knowledge about medication during pregnancy using univariable and multivariable logistic regression.

Variable	Subgroup	count	Univariable Logistic Regression		Multivariable Binary Logistic Regression	
			COR (95% C.I)	P value	AOR (95% C.I)	P value
Age (years)	≤ 25	580	Reference		Reference	
	>25	423	1.65 (1.24–2.20)	0.001*	1.09 (0.77–1.54)	0.644
Education level	high school or less	828	Reference		Reference	
	Diploma or higher	175	6.67 (4.70–9.46)	<0.001*	4.00 (2.64–6.07)	<0.001*
Living place	Rural	265	Reference		Reference	
	Urban	738	2.79 (1.90–4.10)	<0.001*	1.64 (1.07–2.53)	0.024*
Income	≤ 55000 YR	533	Reference		Reference	
	>55000 YR	470	2.46 (1.84–3.29)	<0.001*	1.23 (0.87–1.73)	0.242
Job	Unemployed	905	Reference		Reference	
	Employed	98	2.84 (1.86–4.34)	<0.001*	0.92 (0.53–1.58)	0.758
Insurance	No	890	Reference		Reference	
	Yes	113	3.57 (2.39–5.33)	<0.001*	1.71 (1.07–2.74)	0.026*
Gravidity	≤ 3 times	628	Reference		-	-
	> 3 times	375	0.88 (0.66–1.18)	0.407	-	-
Have you experienced none completed pregnancies	Yes	347	Reference		Reference	
	No	656	0.79 (0.59–1.05)	0.106	0.99 (0.70–1.42)	0.976
Visiting a physician before pregnancy for preconception planning	No	766	Reference		Reference	
	Yes	237	1.60 (1.17–2.20)	0.003*	1.15 (0.79–1.67)	0.467
How often do you visit your gynecologist?	Every 6 weeks	568	Reference		Reference	
	Every 4 weeks or less	434	2.00 (1.50–2.66)	<0.001*	1.23 (0.89–1.71)	0.216
Do you have chronic diseases or any medical issues?	No	706	Reference		Reference	
	Yes	297	0.672 (0.48–0.930)	0.016*	0.75 (0.52–1.09)	0.132
Receiving information about the health risks of medications to the fetus	No	171	Reference		Reference	
	Yes	832	4.04 (2.37–6.91)	<0.001*	1.96 (1.10–3.51)	0.023*
Have you used folic acid in previous pregnancies?	No	434	Reference		Reference	
	Yes	569	2.38 (1.76–3.23)	<0.001*	1.65 (1.14–2.40)	0.008*
Are you currently taking folic acid?	No	346	Reference		Reference	
	Yes	657	6.54 (4.28–9.99)	<0.001*	4.26 (2.71–6.69)	<0.001*
Check the leaflets of the medication	No	666	Reference	Reference		
	Yes	337	5.67 (3.938–8.155)	< .001*	3.29 (2.178–4.964)	< .001*

Abbreviations: YER: Yemeni Riyal (One US Dollar = 550 Yemeni Riyals); COR: Crude odd ratio; AOR: adjusted odd ration; star sign (*) and bold text indicate significant result

In the subsequent multivariable analysis, adjusting for these variables, education level remained a significant predictor of better knowledge (AOR: 4.00, *P* < .001), as did insurance status (AOR: 1.71, *P* = .026), information about medication risks to the fetus (AOR: 1.96, *P* = .023), using of folic acid either in a previous pregnancy (AOR: 1.65, *P* = .008) or in the current pregnancy (AOR: 4.26, *P* < .001), and checking the medication leaflet (AOR: 3.29, *P* < .001).

### Beliefs about taking medication during pregnancy

Pregnant women’s opinions about medication use during pregnancy are summarized in Table 5. A significant proportion disagreed that all medications pose harm to the fetus (54.3%), while the majority agreed that pregnant women should cease medication intake for the benefit of the fetus (58.8%). A substantial majority considered it better for the fetus if medications were used for treatment rather than leaving conditions untreated (71.0%). Furthermore, a notable portion believed that medications during pregnancy save fetal lives annually (65.1%). It was widely agreed that pregnant women should consult healthcare providers before using natural remedies (91.3%) and consult a gynecologist before taking any medication (98.7%). Additionally, a large majority endorsed the discontinuation of unnecessary medications without a prescription during pregnancy (97.2%).

**Table 5:** Participants’ beliefs on taking medication during pregnancy.

Statements	Agree	Uncertain	Disagree
All medications can be harmful to the fetus.	261(26.0)	197(19.6)	545(54.3)
It is best for the fetus if the pregnant woman stops taking medications during pregnancy ^	590(58.8)	159(15.9)	254(25.3)
Pregnant women have a higher limit for medication use compared to non-pregnant women.	335(33.4)	183(18.2)	485(48.4)
It is better for the fetus if pregnant women use medications for treatment instead of leaving the condition untreated ^	711(71.0)	116 (11.6)	175(17.5)
Medications during pregnancy can save the lives of many fetuses every year ^	652(65.1)	284(28.4)	65(6.5)
Physicians frequently prescribe medications to pregnant women.	235(23.5)	262(26.2)	506(50.4)
Natural remedies can generally be used by pregnant women.	202(20.1)	228(22.7)	573(57.1)
Pregnant women should consult any healthcare provider before using natural remedies ^	916(91.3)	29(2.9)	58(5.8)
It is necessary to consult a gynecologist before taking any medication ^	990(98.7)	5(0.5)	8(0.8)
You should stop taking unnecessary medications without a prescription during pregnancy ^	975(97.2)	11(1.1)	17(1.7)
	Type of belief	N = 1003	Percentage
Belief on taking medication during pregnancy (summary index from 6 items)	Negative	761	(75.9)
	Positive	242	(24.1)

### Sources of information

**Fig 1:**
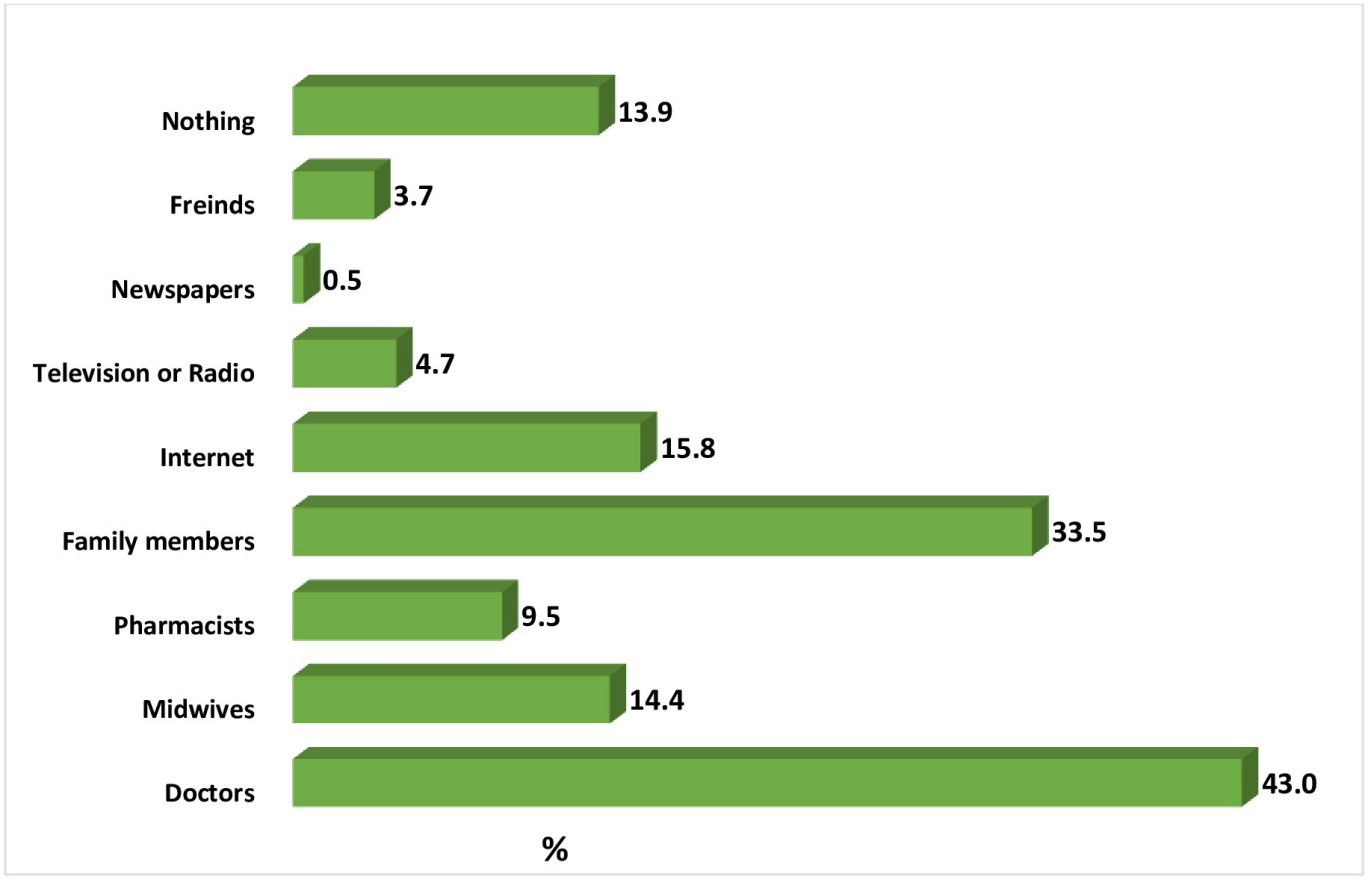
Source of information about medication during pregnancy.

Regarding the source of information about medication during pregnancy, physicians are the primary source (43.0%), followed by family members (33.5%), internet (15.8%), and midwives (14.4%). More details are shown in Fig 1.

### Number of medications utilized during pregnancy and their anatomical therapeutic chemical (ATC) classes

Table 6 shows the analysis of the number of medications utilized during pregnancy and their pharmacological classes according to the WHO’s first level of the ATC classification. The study involved 1,003 participants, shedding light on their medication uses. A total of 2,623 medications were utilized during pregnancy, with the majority being prescribed medications.

**Table 6:** Number of medications utilized during pregnancy and their pharmacological classes according to 1^st^ level of ATC classification (n = 1003).

	**Overall medications (n = 2623)**		**Prescribed-only medication (n = 2316)**		**Non- prescribed medications (n = 307)**	
	N	%	N	%	N	%
First trimester	468	(17.8)	412	(17.8)	56	(18.2)
Second trimester	872	(33.2)	773	(33.4)	99	(32.2)
Third trimester	1283	(48.9)	1131	(48.8)	152	(49.5)
Total	2623	100.0	2316	100.0	307	100.0
	**Overall medications (n = 2636)**		**Prescribed-only medications (n = 2328)**		**OTC medications (n = 308)**	
	N	%	N	%	N	%
A	620	(23.5)	567	(24.4)	53	(17.2)
B	1037	(39.3)	985	(42.3)	52	(16.9)
C	97	(3.7)	96	(4.1)	1	(0.3)
D	6	(0.2)	6	(0.3)	0	(0.0)
G	333	(12.6)	326	(14.0)	7	(2.3)
H	9	(0.3)	9	(0.4)	0	(0.0)
J	196	(7.4)	192	(8.2)	4	(1.3)
L	1	(0.04)	1	(0.04)	-	-
M	22	(0.8)	3	(0.1)	19	(6.2)
N	203	(7.7)	43	(1.8)	160	(51.9)
P	12	(0.5)	11	(0.5)	1	(0.3)
R	86	(3.3)	81	(3.5)	5	(1.6)
S	2	(0.1)	-	-	2	(0.6)
V	12	(0.5)	8	(0.3)	4	(1.3)
Total	2636		2328		308	-

Abbreviations: A: Alimentary Tract and Metabolism; B: Blood and Blood-Forming Organs; C: Cardiovascular System; D: Dermatological; G: Genito-urinary System and Sex Hormones; H: Systemic Hormonal Preparations, Excluding Sex Hormones and Insulins; J: Anti-infectives for Systemic Use; L: Antineoplastic and Immunomodulating Agents; M: Musculo-skeletal System; N: Nervous System; P: Antiparasitic Products, Insecticides, and Repellents; R: Respiratory System; S: Sensory Organs; V: Various

Regarding medication distribution across pregnancy trimesters, the majority of medications (48.9%, n = 1283) were utilized during the third trimester, followed by the second trimester (33.2%, n = 872), and the first trimester (17.8%, n = 468).

In terms of the pharmacological categorization by ATC code level 1, notable variations in medication preferences were highlighted. The largest portion (39.3%, n = 1037) of the used medications belonged to the blood and blood-forming organs (B) category, followed by the alimentary tract and metabolism category (A), accounting for 23.5% (n = 620) of the used medications, and genitourinary system and sex hormones category (G), accounting for 12.6% (n = 333) of the total medications.

## Discussion

To examine knowledge, beliefs, and practices regarding the use of medications during pregnancy and their associated factors, a sample of pregnant women in Yemen was used in this questionnaire. This information is important for guiding interventions and activities by policymakers and healthcare professionals.

Around one-third of the females who participated in this study revealed that they had primary education. It was reported that there is a gender gap caused by socioeconomic, cultural, and religious reasons in Yemen. The current political situation also has a negative effect on education [23]. A national population survey conducted in Yemen that was published in 2008 revealed that 40.9% of women chewed khat during pregnancy [24]. In this questionnaire, more than half the participants were chewing khat despite its risk to maternal and fetal health conditions, as revealed in various studies [25, 26]. However, this did not prevent women from chewing it.

More than 84% of the pregnant women participating in this study reported the use of at least one prescribed medication. This high percentage was comparable to similar studies conducted in Malaysia [16], Scotland [27], the United States [28], and the United Kingdom (UK) [29]. The case with non-prescribed medications was different, as only around a quarter of the participants reported the use of OTC medications during their pregnancy. This percentage is lower than what was reported in similar studies conducted in Italy [21], Europe, North and South America, and Australia [3]. However, the use of OTC medication among the participating pregnant women in Yemen was higher than what was reported in Ireland [30] and the Netherlands [31].

Numerous pregnancy issues, including congenital heart disease, hypertension, intrauterine growth restriction, recurrent miscarriages, placental abruption, and premature labor, were found to be related to folic acid deficiency [32]. Pregnant women are recommended to take 400 *μ*g of folic acid daily, which may be obtained from fortified foods and vitamin supplements, to decrease the risk of neural tube abnormalities by half. In high-risk pregnancies that were previously affected, 400 *μ*g folic acid intake is to start 1–3 months before conception [32]. Around 65% of the participants reported using folic acid during their pregnancy. In Lybia, 73% of women participating in a similar study reported the use of folic acid [33]. The situation in Rawalpindi, Pakistan, was worse as only around half of pregnant women took folic acid [34].

Regarding the knowledge and awareness of medication use during pregnancy, most participants were aware that certain medications might not be safe during pregnancy, while others are important, and they knew the critical period during pregnancy where the use of medications must be restricted. These results are better than what was obtained from a similar study conducted in a hospital in India [35], Malaysia [16], and Italy [21]. Pregnant women were also found to be more cautious regarding using medications in a study conducted in Saudi Arabia [36]. The majority of pregnant women were aware of folic acid as a vitamin supplement; however, only around 18% of them knew that folic acid deficiency is associated with neural tube defects. Similarly, in a study conducted in Libya, nearly three-quarters of the pregnant women were aware of folic acid but had a low overall level of awareness regarding its importance [33]. In a study targeting women of child bearing age in the UK, it was found that knowledge regarding folic acid deficiency was low along with poor attitude [34].

In this study, higher knowledge was associated with older age, higher education, living in urban areas, higher income, employment, insurance, visiting the gynecologist more frequently, preconception planning, suffering from a medical issue, using folic, and checking the medication leaflet. Education was found to be linked with better knowledge regarding folic acid in studies conducted in Pakistan [34], and the UK [29]. Additionally, older women were found to be more knowledgeable about medication use in pregnant women in Southern Italy [21]. In the same study, other factors that were linked to better knowledge were found to be employed, with no history of abortion, having a medical problem in the previous year, and having a better self-perceived health status [21]. A study conducted in Malaysia found similar findings regarding the association between older age and being pregnant before with higher knowledge regarding medication use during pregnancy [16]. Moreover, age, education, occupation, and area of living were the factors influencing the knowledge of pregnant women on medication use in a similar study conducted in India [35].

Regarding the participants’ beliefs, the majority agreed that pregnant women should cease medication intake for the benefit of the fetus, but only around 26% thought that all medicines could be harmful to the fetus. However, in a study conducted in Saudi Arabia, 59.2% of the women believed that all medicines could be harmful to the fetus [36]. In India, 35.55% of the women thought it was best for the developing fetus if pregnant women stopped taking all medication [35]. In Malaysia, 56.5% of the women had negative beliefs about medication use during pregnancy [16]. Additionally, in this study, 71% of the women believed that it is better for the fetus if pregnant women use medications for treatment instead of leaving the condition untreated. This percentage is higher than what was found in Saudi Arabia, the neighboring country to Yemen, where only 44.7% had a similar belief [36]. The same is noted in believing that using medications during pregnancy can save the lives of many fetuses every year, as 65.1% of this study participants agreed with this statement, while only 46.1% of Saudi women believed so [36]. However, similar to the findings of the Saudi study [36], women in Yemen did not have a strong belief that natural products are safer for pregnant women. Luckily, the majority of Yemeni women participating in this study believed that they should consult their doctors and gynecologists before taking any medication or natural products.

For the participating women in this study, physicians and family members were the main sources of information regarding medication use during pregnancy. Saudi women, unlike Yemeni women, relied on gynecologists, general practitioners, and pharmacists for information regarding medication use during pregnancy [36]. Physicians, pharmacists, and gynecologists were also the main sources of information for Malaysian women [16]. Surprisingly, in the UK, 50% of women used the internet to get information about medication use during their pregnancies [29].

It was found in this study that most of the medications used during pregnancy were prescribed, with the largest portion belonging to the blood and blood-forming organs, followed by the alimentary tract, genitourinary system, and sex hormones. In Europe, North and South America, and Australia, medications to treat hypothyroidism, asthma, allergy, and depression were the most commonly used prescribed medications [3]. In the same study, it was found that there was substantial inter-region variability in the types of medication used [3].

This study possesses several notable strengths. Firstly, it addresses a timely and relevant issue by investigating the increasing use of medications among pregnant women. Secondly, it’s a multi-center approach involving four governorates in Yemen, enhancing the study’s representativeness and increasing the reliability of the findings. Furthermore, the application of both univariable and multivariate logistic regressions allows for a comprehensive analysis of factors influencing pregnant women’s knowledge about medication use, providing valuable insights for future interventions.

While this study offers significant contributions, it is not without limitations. First, its cross-sectional design, while providing valuable insights, only offers a snapshot of the study population at a specific point in time, limiting its ability to establish causal relationships or track changes in knowledge and practices over time. Second, the study’s sample primarily consists of pregnant women seeking antenatal care at tertiary care hospitals, potentially excluding individuals with different healthcare-seeking behaviors or those residing in areas where only midwives are available. This may limit the generalizability of the findings to a broader population. Third, the reliance on self-reported data, including medication use and knowledge, may introduce the possibility of recall bias and social desirability bias, where participants may provide socially acceptable responses.

## Conclusion

The findings underscore the pressing need for educational interventions aimed at pregnant women. These interventions must address not only the potential risks associated with medication use for both maternal health and fetal development but also emphasize the significance of appropriate medication when needed. By bridging knowledge gaps and promoting informed decision-making, such initiatives can contribute to a safer and healthier pregnancy journey, reducing the inclination towards self-medication.

## Supporting information

S1 File Data collection form. (PDF)

S2 File Raw data. (XLSX)
