# Supplementary material for: Insights and perceptions: Investigating pregnant women’s attitudes, understanding, and factors influencing knowledge regarding medication usage during pregnancy—A cross-sectional study
Source: PLoS One. 2024 Oct 1;19(10):e0311235. doi: 10.1371/journal.pone.0311235 (PMC11444415; doi:10.1371/journal.pone.0311235)
Supplement: S1 File — (PDF) [file pone.0311235.s001.pdf]

QUESTIONNAIRE

Interview date: ………… Number: ………… Hospital: ………… Clinic: …………..

Socio-demographic features:						
Age (in years)	20 -18 ☐	25 -21 ☐	30-26 ☐	35-31 ☐	40 -36 ☐	40< ☐
Education level	☐ Illiterate<br>☐ Primary<br>☐ Secondary<br>☐ High school<br>☐ Diploma or higher					
Living place:<br>☐ Urban<br>☐ Rural	Governorate :<br>☐ Sana'a<br>☐ Dhamar<br>☐ Ibb<br>☐ Al bayda					
Nationality	☐ Yemeni ☐ not-Yemeni					
Income/month in Yemeni riyals	☐ < 55,000<br>☐ 55,000 - < 1,100,000<br>☐ 110,000 - < 165,000<br>☐ 165,000 - < 220,000<br>☐ > 220,000					
Job	☐ Employed ☐ Unemployed					
Smoking before pregnancy	☐ Yes ☐ no<br>If the answer is "Yes", ask her if she smoked at any time during her current pregnancy:<br>☐ Yes ☐ no					
Khat chewing before pregnancy	☐ Yes ☐ no<br>If the answer is "Yes", ask her if she chewed khat during this pregnancy:<br>☐ Yes ☐ no					
Do you have any health insurance?	☐ Yes ☐ no					
Number of pregnancies (including the current pregnancy)	…………………………………..					
How many births have you had before?	…………………………………..					
Have you experienced none completed pregnancies?	☐ Yes ☐ no<br>If yes, how often? ____________ What week did it happen?					
The current gestational age (i.e. 1 week)	……………					
Did you visit your doctor before pregnancy for preconception planning?	☐ Yes ☐ no					
Do you have chronic diseases or any medical issues?	☐ Yes ☐ no					

Diabetes	☐Yes ☐no	Hypertension	☐Yes ☐no
Kidney disease	☐Yes ☐no	Lipid disorders	☐Yes ☐no
Peptic ulcer	☐Yes ☐no	sister	☐Yes ☐no
Heart Failure	☐Yes ☐no	Arrhythmia	☐Yes ☐no
Hypothyroidism	☐Yes ☐no	Asthma or chronic obstructive pulmonary disease	☐Yes ☐no
Hyperthyroidism	☐Yes ☐no	Anemia	☐Yes ☐no

Diseases resulting from arterial problems, such as: myocardial infarction (MI), stable or unstable angina, stroke, transient ischemic attack, stenting, coronary artery bypass graft (CABG) surgery	☐Yes ☐no
other	Please write any other diseases the patient suffers from here:
How often do you visit your gynecologist?<br>□ every two weeks<br>□ every 4 weeks<br>□ every 6 weeks<br>□ every 8 weeks<br>□ more than 8 weeks	
Before pregnancy, do you usually check the medication leaflet for information about the medications you use?	

□Yes □no □sometimes	
During pregnancy, do you usually check the leaflets and safety information of the medication you use?<br>□ Yes □ No	

B. Medication use during pregnancy

I will ask you some questions to gather information about the medications you are using during this pregnancy.

Q: Do you inform the pharmacist that you are pregnant when purchasing any medication from the pharmacy?

□Yes □no

If no, what are the reasons ( )

Q: Do you take any prescription-only medication in the current pregnancy??

□ yes □no

Use of prescribed medications			
Medicine	What is the dosing regimen and how long have you been using the medication?	For any health problem?	Did you start this treatment before or during pregnancy?(select week)
1			
2			
3			
4			
5			
6			

Q: Have you used any non-prescribed medications during the current pregnancy? (medicines that are dispensed without a prescription, such as those used for the following conditions:sore throat, minor burns, headache, cough, colds, nasal decongestant medication, nausea, vomiting, diarrhea, constipation, toothache, indigestion, heartburn, minor pain, discomfort and fever (such as aches and sprains, headaches, menstrual pain, Back pain)?

□ yes □no

Use of non-prescription medications (e.g.: acetaminophen, aspirin, antacids, decongestants, antihistamines, laxatives, etc...), as well as folic acid, multivitamins, and iron.)			
What medication did you use during or during pregnancy?	What is the dosing regimen and how long have you been using the medication?	For what health condition?	Did you start this treatment before or during pregnancy?<br>(Specify the week if during pregnancy)
1			
2			
3			
4			
5			
6			

c. General knowledge about medication use during pregnancy

I will ask you some questions to explore your knowledge regarding medication use during pregnancy:

1. Are you aware that some medications may not be safe during pregnancy?
   □Yes □ No
2. Are you aware that some medications are important during pregnancy?
   □Yes □ No
3. Do you know what is the critical period during pregnancy, where medications may have a greater impact on the fetus?
   □Yes □ No

   If the answer is yes to the previous question, please specify the time period

   A. The first 3 months
   B. 4-6 Months
   C. 7-9 months
4. Is it recommended to adjust medications for chronic conditions before pregnancy?
   □ yes □no

Knowledge of folic acid:

1.	Have you heard of folic acid?	□Yes □no
2.	Are you aware of the role of folic acid during pregnancy?	□Yes □no<br>If she answers that it prevents deformation, consider it a yes
3.	Have you heard about neural tube defects?	□Yes □no
4.	Is there a connection between folic acid deficiency and congenital diseases?	□Yes □no
5.	What is the appropriate time to start taking folic acid supplements during pregnancy?	□Before pregnancy (during pregnancy planning)
		□At the beginning of pregnancy (in the first four weeks of pregnancy)
		□First trimester (first to third month)
		□Second trimester (fourth to sixth month)
		□Third trimester (seventh to ninth months)
		□I don't know (if a doctor prescribes it, I take it)
6.	In your current pregnancy, when did you start using folic acid?	□Before pregnancy (during pregnancy planning)
		□In early pregnancy (after you find out you're pregnant)
		□First trimester of pregnancy (first to third months)
		□Second trimester (fourth to sixth month)
		□Third trimester (seventh to ninth months)
		□I still don't use it
7.	Have you used folic acid in previous pregnancies?	□Yes □no □I do not remember □I don't know
8.	Are you currently taking folic acid?	□Yes □no
9.	What is the recommended prophylactic dose of folic acid for pregnant women without any other health conditions?	□ 1 mg(1000mcg)<br>□ 5 mg(5000mcg)<br>□ (400-600 µg)

D- Beliefs about medications used during pregnancy

I will ask you some questions to gather information about your attitudes towards medications during pregnancy

	Statements	Agree	Uncertain	Disagree
1	All medications can be harmful to the fetus.			
2	It is best for the fetus if the pregnant woman stops taking medications during pregnancy			
3	Pregnant women have a higher limit for medication use compared to non-pregnant women.			
4	It is better for the fetus if pregnant women use medications for treatment instead of leaving the condition untreated			
5	Medications during pregnancy can save the lives of many fetuses every year			
6	Physicians frequently prescribe medications to pregnant women.			
7	Natural remedies can generally be used by pregnant women.			
8	Pregnant women should consult any healthcare provider before using natural remedies			
9	It is necessary to consult a gynecologist before taking any medication			
10	You should stop taking unnecessary medications without a prescription during pregnancy			

Information sources

I'm going to ask you some questions to find out the sources and need for information about medications used in pregnancy.
From what sources did you receive information about medications during pregnancy? (more than one answer if possible)

□Doctors
□ Midwives
□ Pharmacists
□ Internet
□ Friends
□ Family members
□ Television and Radio
□ Newspapers
□ nothing
□ Other: specify:
